# Antithrombin attenuates myocardial dysfunction and reverses systemic fluid accumulation following burn and smoke inhalation injury: a randomized, controlled, experimental study

**DOI:** 10.1186/cc12712

**Published:** 2013-05-11

**Authors:** Sebastian Rehberg, Yusuke Yamamoto, Eva Bartha, Linda E Sousse, Collette Jonkam, Yong Zhu, Lillian D Traber, Robert A Cox, Daniel L Traber, Perenlei Enkhbaatar

**Affiliations:** 1 Investigational Intensive Care Unit, Department of Anesthesiology, The University of Texas Medical Branch, 301 University Blvd., Galveston, TX 77555, USA; 2 Department of Anaesthesiology and Intensive Care, University of Muenster, Albert-Schweitzer-Campus 1, 48149 Muenster, Germany; 3 Department of Pathology, The University of Texas Medical Branch, 301 University Blvd. 33, Galveston, TX 77555, USA; 4 Shriners Hospital for Children, 815 Avenue D, Galveston, TX 77550, USA

**Keywords:** capillary leakage, cardiovascular hemodynamics, mitogen-activated protein kinase, myocardial oxygen consumption, tumor necrosis factor, left ventricular dysfunction

## Abstract

**Introduction:**

We hypothesized that maintaining physiological plasma levels of antithrombin attenuates myocardial dysfunction and inflammation as well as vascular leakage associated with burn and smoke inhalation injury. Therefore, the present prospective, randomized experiment was conducted using an established ovine model.

**Methods:**

Following 40% of total body surface area, third degree flame burn and 4 × 12 breaths of cold cotton smoke, chronically instrumented sheep were randomly assigned to receive an intravenous infusion of 6 IU/kg/h recombinant human antithrombin (rhAT) or normal saline (control group; *n *= 6 each). In addition, six sheep were designated as sham animals (not injured, continuous infusion of vehicle). During the 48 h study period the animals were awake, mechanically ventilated and fluid resuscitated according to standard formulas.

**Results:**

Compared to the sham group, myocardial contractility was severely impaired in control animals, as suggested by lower stroke volume and left ventricular stroke work indexes. As a compensatory mechanism, heart rate increased, thereby increasing myocardial oxygen consumption. In parallel, myocardial inflammation was induced via nitric oxide production, neutrophil accumulation (myeloperoxidase activity) and activation of the p38-mitogen-activated protein kinase pathway resulting in cytokine release (tumor necrosis factor-alpha, interleukin-6) in control vs. sham animals. rhAT-treatment significantly attenuated these inflammatory changes leading to a myocardial contractility and myocardial oxygen consumption comparable to sham animals. In control animals, systemic fluid accumulation progressively increased over time resulting in a cumulative positive fluid balance of about 4,000 ml at the end of the study period. Contrarily, in rhAT-treated animals there was only an initial fluid accumulation until 24 h that was reversed back to the level of sham animals during the second day.

**Conclusions:**

Based on these findings, the supplementation of rhAT may represent a valuable therapeutic approach for cardiovascular dysfunction and inflammation after burn and smoke inhalation injury.

## Introduction

Burn- and smoke inhalation-induced shock not only implies hemodynamic instability and vascular leakage but also severe myocardial dysfunction [1], which already occurs two hours after the injury [2]. Contrary to septic cardiomyopathy [3], for example, the clinical relevance of burn- and smoke-induced myocardial dysfunction is only poorly recognized. However, there is a significant correlation between left ventricular dysfunction and in-hospital mortality of critically burned patients [4]. In addition, myocardial stress caused by burn injuries persists for up to three years, thereby also influencing long-term outcome [5]. Therefore, effective treatment strategies for burn- and smoke-induced myocardial dysfunction are warranted.

The proposed pathogenesis of burn- and smoke-induced myocardial dysfunction is based on inflammatory mechanisms, including activation of the p38-mitogen-activated protein kinase (p38-MAPK) pathway as well as production of tumor necrosis factor alpha (TNF-α) and nitric oxide (NO) [6-8]. The plasma-derived glycoprotein antithrombin III (AT) has been shown to reduce neutrophil activation [9] and to attenuate all of these inflammatory cascades in lung injury, for example [10-12]. However, potential benefits of AT on cardiovascular inflammation and dysfunction following burns and smoke inhalation have not been investigated, yet. A second rationale for the use of AT in these patients is an AT deficiency following burn injuries [13], that represents an independent predictor of length of hospital stay and mortality [14,15].

Accordingly, we hypothesized that maintaining physiological plasma levels of AT reduces myocardial inflammation and attenuates cardiovascular dysfunction associated with burn and smoke inhalation injury. Therefore, the present prospective, randomized, controlled laboratory experiment was designed to elucidate the effects of an intravenous infusion of recombinant human AT (rhAT) on myocardial neutrophil accumulation, activation of the p38-MAPK pathway, myocardial TNF-α secretion and systemic NO production as well as cardiovascular hemodynamics and systemic fluid accumulation in an established ovine model [16,17].

## Materials and methods

The study was approved by the Animal Care and Use Committee of the University of Texas Medical Branch at Galveston and conducted in compliance with the guidelines of the National Institutes of Health.

### Instrumentation and surgical procedures

Eighteen female sheep were anesthetized and instrumented for chronic hemodynamic monitoring using an established protocol [16,17]. Details are provided in Additional file 1, Materials and methods.

### Experimental protocol

Following baseline measurements (BL) in the healthy sheep, a tracheostomy was performed and a urinary bladder catheter was placed under deep anesthesia. Twelve animals were then subjected to 40% of total body surface area third degree flame burn and 4 × 12 breaths of cold cotton smoke under deep anesthesia using an established protocol [16,17]. The arterial carboxyhemoglobin level was determined immediately after smoke inhalation to quantify the degree of injury. Although third degree burns are considered painless, 0.03 mg buprenorphine i.v. was administered before the burn and every 12 h subsequently to provide analgesia for the edges of the burn area, which may be second-degree burns. The sheep were then randomly assigned to the control group (injured, continuous infusion of vehicle (NaCl 0.9%), *n *= 6) or the rhAT group (injured, continuous infusion of 6 IU/kg/h rhAT (GTC Biotherapeutics Inc., Framingham, MA, USA) from 1 h post injury until the end of the 48-h study period, *n *= 6). During the experiment the investigators were unaware of the animal's group assignment. Six animals were assigned to the sham group (not injured, continuous infusion of vehicle (NaCl 0.9%)). Due to the obvious lack of burn injury, this group assignment could not be blinded.

All animals were mechanically ventilated with a tidal volume of 15 mL/kg and a positive end-expiratory pressure of 5 cmH_2_O throughout the entire experiment. In this context, it is important to consider the study design and the physiological differences between sheep and humans (see in detail Additional file 1, Materials and methods). The inspiratory oxygen fraction was set at 100% for the first three hours post injury, and was then adjusted to maintain oxygenation (arterial oxygen saturation >90%, partial pressure of oxygen >90 mmHg), whenever possible. The respiratory rate was adjusted according to individual blood gas analyses to ensure normocapnia.

Resuscitation was performed with lactated Ringer's solution according to the Parkland formula (4 mL/kg/% burned body surface area within 24 h) [18]. To compensate for the initial fluid loss, sheep received one half of the total calculated amount for 24 h within the first 8 h after injury. At the end of the 48-h study period, sheep were deeply anesthetized with ketamine (15 mg/kg) and killed by injection of 60 mL of saturated potassium chloride.

### Hemodynamic monitoring and laboratory analyses

Hemodynamic measurements, analyses of blood samples for gas tensions, plasma levels of AT and NO, protein concentration, oncotic pressures, variables of plasmatic coagulation and creatinine (plasma and urine) were performed at specific time points. Details are provided in Additional file 1, Materials and methods.

### Immunohistochemical analysis and Western blots

Samples from the left ventricle (anterior wall) were used for quantification of myeloperoxidase activity, p38-MAPK, TNF-α and interleukin-6 (IL-6). Myeloperoxidase activity was determined using a commercially available assay (Myeloperoxidase Activity Assay, Northwest Life Science Specialties, Vancouver, BC, Canada) according to the manufacturer's protocol. Details are provided in Additional file 1, Materials and methods.

### Statistical analyses

Sigma Stat 3.1 software (Systat Software, Inc., San Jose, CA, USA) was used for statistical analyses. Analysis of variance on ranks methodologies appropriate for non-normally distributed variables with repeated measures were used. Each variable was analyzed separately for differences among groups, differences across time, and for group by time interaction. After confirming the significance of different group effects over time, *post hoc *pair-wise comparisons among groups were performed using the Student-Newman-Keuls procedure to adjust for the elevated false positive rate found otherwise in multiple testings. Finally, a rank sum test was applied to compare values at each time point. Western blot analyses and myeloperoxydase activity were compared with the rank sum test. Data are expressed as median with interquartile range (25^th^; 75^th^). Differences were considered as statistically significant when *P *was less than 0.05.

## Results

### Baseline characteristics

The mean body weight of the sheep was 33 kg (29; 38). There were no differences among study groups in any of the investigated variables at BL. Carboxyhemoglobin values after smoke inhalation, as an index of the severity of injury, did not differ among groups (control: 69% (58; 74) vs. rhAT: 73% (58; 80), *P *= 0.610).

### Cardiovascular hemodynamics

**Figure 1:**
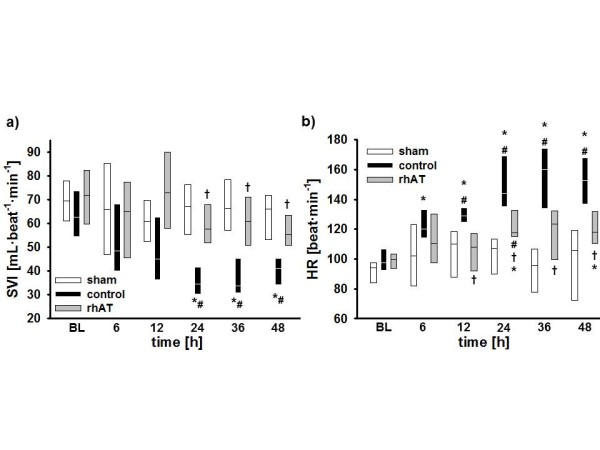
**Stroke volume index (a) and heart rate (b)**. *P <0.05 vs. baseline; ^#^*P *<0.05 vs. sham; ^† ^*P *<0.05 vs. control; data are represented as box plots with median and interquartile range (25^th^; 75^th^); *n *= 6 per group. BL, baseline; HR, heart rate; rhAT, recombinant human antithrombin III; SVI, stroke volume index

**Figure 2:**
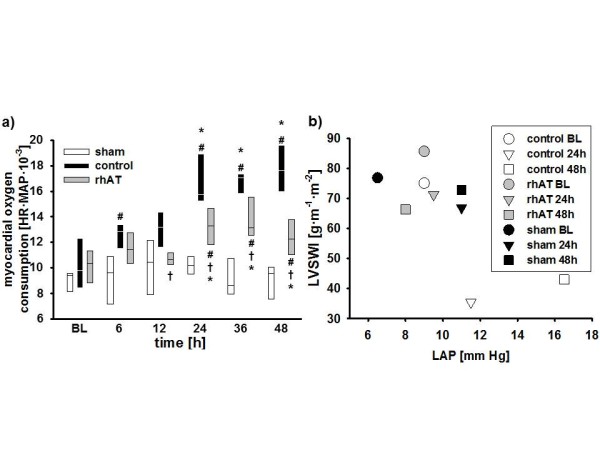
**Myocardial oxygen consumption (a) and left ventricular contractility (b)**. Data are represented as box plots with median and interquartile range (25^th^; 75^th^) (a) or median (b); *n *= 6 per group. BL, baseline; HR, heart rate; LAP, left atrial pressure; LVSWI, left ventricular stroke work index; MAP, mean arterial pressure; rhAT, recombinant human antithrombin III.

Stroke volume index (SVI; 24 to 48 h: *P *<0.05 vs. BL and 24 to 48 h: *P *<0.05 vs. sham, Figure 1a) and left ventricular stroke work index (LVSWI, 12 to 48 h: *P *<0.05 vs. BL and 24 to 48 h: *P *<0.05 vs. sham, Table 1) decreased by about 50% vs. BL in the control group within 24 h. In these animals, cardiac index was maintained (no statistical differences vs. BL) by a progressive increase in heart rate (HR; 6 to 48 h: *P *<0.05 vs. BL and 12 to 48 h: *P *<0.001 vs. sham, Figure 1b). Myocardial oxygen consumption [19] increased significantly (24 to 48 h: *P *<0.05 vs. BL and 6 h, 24 to 48 h: *P *<0.05 vs. sham, Figure 2a). rhAT treatment attenuated the decreases in SVI (24 to 48 h: *P *<0.05 vs. control) and LVSWI (24 to 48 h: *P *<0.05 vs. control each), resulting in a lower HR and oxygen consumption than in control animals (12 to 48 h: *P *<0.05 each). With one exception at 24 h (rhAT and sham vs. control: *P *<0.05 each), there were no significant differences in cardiac index between study groups (Table 1). Right ventricular stroke work index was lower in control animals as compared to sham from 24 to 36 h (*P *<0.05 each). Left atrial pressure (LAP) increased over time in the control group (24 to 48 h: *P *<0.05 vs. BL each). rhAT infusion was associated with a lower LAP (48 h: *P *= 0.016 vs. control). Similarly, central venous pressure (CVP) increased in control animals (24 to 36 h: *P *<0.05 vs. BL; *P *<0.05 vs. sham), whereas CVP did not increase in rhAT-treated animals (*P *= 0.01 vs. control at 36 h). Left ventricular contractility, expressed as a correlation of LVSWI and LAP, an index of left ventricular preload (Figure 2b), was markedly impaired in the control group, whereas contractility in rhAT-treated animals was comparable to sham animals.

**Table 1:** Variables of cardiovascular hemodynamics

Variable	Time point	sham	control	rhAT
**CI**	BL	6.1 (5.6; 6.7)	5.9 (5.7; 6.7)	6.8 (6.1; 7.8)
**(L/min/m^2^)**	6 h	6.7 (6.0; 7.5)	6.2 (5.3; 7.2)	6.7 (5.6; 7.5)
	12 h	6.9 (6.4; 7.0)	6.0 (4.7; 7.4)	7.8 (6.9; 8.4)
	24 h	7.1 (6.6; 7.4)	5.5 (5.0; 5.6)^#^	8.0 (6.8; 8.8)^† ^
	36 h	6.2 (5.4; 7.3)	5.6 (5.3; 6.4)	6.7 (6.3; 7.8)
	48 h	6.5 (6.4; 6.9)	4.5 (4.4; 7.2)	6.7 (5.8; 7.7)
**MAP**	BL	97 (93; 98)	101 (93; 113)	105 (96; 110)
**(mmHg)**	6 h	100 (94; 102)	103 (99; 108)	104 (96; 108)
	12 h	95 (91; 104)	100 (98; 109)	102 (94; 102)
	24 h	92 (89; 98)	102 (99; 107)	95 (93; 106)
	36 h	92 (87; 98)	100 (89; 104)	103 (100; 108)
	48 h	93 (90; 98)	101 (90; 111)	101(99; 106)
**CVP**	BL	5 (5; 5)	6 (5; 8)	5 (4; 6)
**(mmHg)**	6 h	7 (6; 11)	9 (7; 10)	9 (8; 10)
	12 h	9 (5; 10)	10 (7; 11)	9 (7; 9)
	24 h	8 (7; 10)	12 (10; 14)*^#^	7 (5; 11)
	36 h	10 (7; 11)	13 (12; 14)*^#^	7 (6; 10)^† ^
	48 h	9 (9; 11)	11 (7; 13)	6 (5; 8)
**LAP**	BL	7 (6; 8)	9 (8; 10)	9 (9; 10)
**(mmHg)**	6 h	9 (9; 9)	9 (7; 11)	10 (8; 12)
	12 h	12 (11; 12)	11 (9; 13)	11 (9; 12)
	24 h	11 (10; 12)	12 (11; 13)*	10 (9; 11)
	36 h	12 (9; 13)	14 (13; 16)*	10 (8; 14)
	48 h	11 (9; 13)	17 (15; 18)*^#^	8 (7; 9)^† ^
**LVSWI**	BL	77 (67; 87)	75 (67; 83)	86 (73; 93)
**(g/m/m^2^)**	6 h	78 (57; 89)	57 (45; 75)	79 (64; 86)
	12 h	66 (64; 72)	50 (44; 60)*	74 (60; 91)
	24 h	67 (59; 77)	35 (34; 37)*^#^	71 (56; 80)^† ^
	36 h	71 (65; 76)	35 (31; 43)*^#^	73 (61; 86)^† ^
	48 h	73 (72; 76)	43 (42; 55)*^#^	66 (63; 73)^† ^
**RVSWI**	BL	13 (13; 14)	12 (10; 15)	13 (11; 15)
**(g/m/m^2^)**	6 h	13 (11; 17)	13 (10; 15)	13 (11; 17)
	12 h	16 (14; 17)	13 (11; 16)	16 (14; 18)
	24 h	15 (15; 17)	9 (8; 11)^#^	15 (11; 17)
	36 h	16 (14; 17)	8 (7; 12)^#^	13 (13; 17)

	48 h	15 (13; 16)	10 (9; 12)	16 (11; 19)

*, *P *<0.05 vs. BL; ^#^, *P *<0.05 vs. sham; ^† ^, *P *<0.05 vs. control; data are represented as median with interquartile range (25^th^; 75^th^); each group *n *= 6

BL, baseline; CI, cardiac index; CVP, central venous pressure; LAP, left atrial pressure; LVSWI, left ventricular stroke work index; MAP, mean arterial pressure; rhAT, recombinant human antithrombin III; RVSWI, right ventricular stroke work index.

### Vascular leakage and fluid accumulation

**Figure 3:**
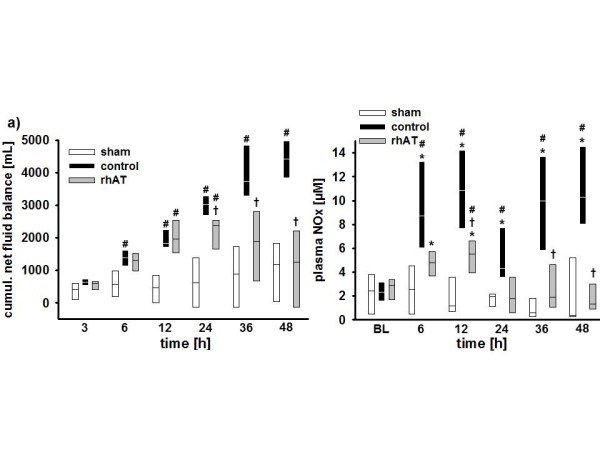
**Cumulative net fluid balance (a) and concentration of NOx in the plasma (b)**. *P <0.05 vs. baseline; ^#^*P *<0.05 vs. sham; ^† ^*P *<0.05 vs. control; data are represented as box plots with median and interquartile range (25^th^; 75^th^); *n *= 6 per group. BL, baseline; NOx, nitrates and nitrites; rhAT, recombinant human antithrombin III.

Vascular leakage was evidenced in control animals by an increased hematocrit (36 h: *P *= 0.017 vs. BL and *P *= 0.048 vs. sham, Table 2) in parallel with decreases in plasma protein concentration and plasma oncotic pressures (6 to 48 h: *P *<0.05 vs. BL and 6 to 48 h: *P *<0.05 vs. sham, Table 2). Accordingly, cumulative net fluid balance progressively increased resulting in a systemic fluid accumulation of about 4,000 mL in the control group at 48 h (12 to 48 h: *P *<0.05 vs. sham, Figure 3a). In rhAT-treated animals, there was an initial decrease in plasma protein concentration and plasma oncotic pressures without any further progression afterwards (*P *<0.01 vs. BL and *P *<0.05 vs. sham each). Hematocrit did not change compared with BL or sham animals in the rhAT group. Cumulative net fluid balance initially increased in rhAT-treated animals from 12 to 24 h (*P *<0.05 vs. sham), but came back to sham level at 48 h (*P *= 0.004 vs. control, Figure 3a).

**Table 2:** Hematocrit, plasma protein concentration, oncotic pressure, urine flow, creatinine clearance and antithrombin plasma levels

Variable	Time point	sham	control	rhAT
**Hematocrit**	BL	27 (22; 29)	24 (23; 27)	26 (21; 26)
**(%)**	6 h	23 (22; 26)	27 (23; 29)	25 (21; 26)
	12 h	26 (23; 30)	28 (24; 31)	26 (24; 27)
	24 h	25 (24; 27)	30 (27; 32)	27 (22; 27)
	36 h	23 (23; 26)	29 (28; 36)*^#^	27 (27; 28)
	48 h	22 (21; 25)	27 (24; 35)	26 (24; 29)
**Protein**	BL	6.1 (6.0; 6.2)	6.2 (5.8; 6.6)	6.1 (6.0; 6.4)
**(g/dL)**	6 h	5.7 (5.6; 6.0)	4.9 (4.6; 5.0)*^#^	5.0 (4.6; 5.4)*^#^
	12 h	6.1 (5.9; 6.4)	4.5 (4.4; 4.6)*^#^	4.9 (4.8; 5.0)*^#† ^
	24 h	5.9 (5.8; 6.2)	4.0 (4.0; 4.0)*^#^	4.6 (4.6; 4.6)*^#† ^
	36 h	5.9 (5.7; 6.2)	3.6 (3.6; 3.9)*^#^	4.7 (4.6; 5.0)*^#† ^
	48 h	5.6 (5.6; 5.8)	3.8 (3.5; 3.9)*^#^	5.0 (4.5; 5.3)*^#† ^
**Oncotic pressure**	BL	19 (17; 20)	19 (18; 20)	20 (19; 22)
**(cmH_2_O)**	6 h	18 (16; 19)	13 (12; 14)*^#^	14 (13; 16)*^#^
	12 h	18 (16; 20)	12 (11; 13)*^#^	14 (13; 15)*^#^
	24 h	19 (16; 20)	10 (9; 10)*^#^	13 (12; 14)*^#† ^
	36 h	19 (16; 20)	9 (9; 11)*^#^	13 (13; 15)*^#† ^
	48 h	17 (15; 18)	9 (9; 10)*^#^	13 (12; 15)*^#† ^
**Urine flow**	BL	/	/	/
**(mL/kg/h)**	6 h	7.7 (6.8; 8.3)	3.4 (2.5; 4.3)^#^	3.2 (1.6; 4.7)^#^
	12 h	7.4 (4.5; 9.0)	2.7 (2.0; 4.5)^#^	2.7 (2.4; 3.8)^#^
	24 h	4.5 (2.7; 5.7)	1.2 (1.1; 2.0)^#^	4.0 (3.0; 5.2)^† ^
	36 h	2.3 (2.0; 2.9)	0.6 (0.3; 0.8)^#^	4.1 (3.7; 5.4)^#† ^
	48 h	2.4 (2.1; 2.5)	0.8 (0.8; 0.9)^#^	3.3 (2.6; 3.8)^† ^
**Creatinine clearance**	BL	/	/	/
**(mL/min)**	6 h	/	/	/
	12 h	105 (97; 129)	89 (78; 141)	78 (76; 124)
	24 h	88 (78; 176)	84 (71; 94)	100 (91; 115)
	36 h	107 (84; 135)	104 (74; 122)	120 (106; 134)
	48 h	102 (81; 112)	91 (88; 94)	96 (82; 115)
**AT plasma level**	BL	86 (83; 89)	79 (72; 82)	87 (73; 93)
**(mg/L)**	6 h	86 (81; 89)	58 (57; 59)*^#^	88 (83; 89)^† ^
	12 h	87 (83; 91)	52 (50; 55)*^#^	85 (81; 95)^† ^
	24 h	86 (73; 88)	45 (40; 50)*^#^	83 (75; 84)^† ^
	36 h	86 (76; 88)	47 (40; 48)*^#^	86 (80; 87)^† ^

	48 h	83 (75; 88)	52 (47; 54)*^#^	84 (73; 87)^† ^

*, *P *<0.05 vs. BL; ^#^, *P *<0.05 vs. sham; ^† ^, *P *<0.05 vs. control; data are represented as median with interquartile range (25^th^; 75^th^); each group *n *= 6

AT, antithrombin; BL, baseline; rhAT, recombinant human antithrombin III

Urine flow in control animals progressively decreased over the study period and was lower than in the sham group (6 to 48 h: *P *<0.05 each, Table 2). In the rhAT group, urine flow was lower than in sham animals from 6 to 12 h (*P *<0.05 each), but higher than in control animals from 24 to 48 h (*P *<0.05 each).

### Gas exchange and global oxygen transport

Arterial oxygen saturation did not differ from BL in sham and rhAT-treated animals over the 48-h study period. In the control group arterial oxygen saturation was lower than BL and sham animals from 24 to 48 h (*P *<0.05 each). Variables on pulmonary gas exchange and ventilation are depicted in detail in Additional file 2, Table S1. There were no persistent differences in variables of global oxygen transport between the study groups (see Additional file 2, Table S1).

### Laboratory analyses

In control animals burn and smoke inhalation was associated with an immediate decrease of AT plasma concentrations (6 to 48 h: *P *<0.05 vs. BL) resulting in lower values than in sham animals (6 to 48 h: *P *<0.05 each, Table 2). The continuous infusion of rhAT prevented this decrease and kept AT plasma concentrations at BL level and higher than in control animals (6 to 48 h: *P *<0.05 each).

No significant differences in activated clotting time, activated partial thromboplastin time and platelet count could be shown over time or between study groups. There was an increase in prothrombin time in both injured groups as compared to BL (36 to 48 h: *P *<0.05) and sham animals (12 to 48 h: *P *<0.05); however, without statistical differences between the control and the rhAT group (see Additional file 2, Table S1).

There were no significant differences between study groups in creatinine clearance during the study period (Table 2).

In control animals, plasma levels of nitrates and nitrites (NOx) increased five-fold within 12 h (6 to 12 h: *P *<0.05 vs. BL and sham each). Then, NOx plasma levels decreased from 12 to 24 h, before they increased again from 24 to 48 h (*P *<0.05 vs. BL and sham each). rhAT infusion attenuated the first increase (12 h: *P *= 0.032 vs. control) and prevented the second increase of plasma NOx compared with control animals (36 to 48 h: *P *<0.05 vs. control, Figure 3b).

### Immunohistochemical analyses and Western blots

Immunohistochemical analyses of myocardial tissues revealed a significant increase in myeloperoxydase activity in control vs. sham animals (*P *= 0.026, Table 3). In rhAT-treated animals, myeloperoxidase activity was lower than in the control group (*P *= 0.041).

**Table 3:** Immunohistochemical analyses and Western blots

Variable	sham	control	rhAT
**Myeloperoxidase activity (U/mL)**	105 (104; 106)	132 (120; 139)^#^	114 (105; 118)^† ^
**p38-MAPK (arbitrary units)**	2.3 (1.8; 3.5)	6.1 (5.4; 6.5)^#^	2.8 (2.3; 3.0)^† ^
**TNF-α (arbitrary units)**	2.1 (1.8; 2.1)	2.7 (2.6; 2.8)^#^	2.0 (1.6; 2.4)^† ^

**IL-6 (arbitrary units)**	1.4 (1.3; 2.0)	4.0 (3.0; 4.6)^#^	2.1 (1.9; 2.8)^† ^

^#^, *P *<0.05 vs. sham; ^† ^, *P *<0.05 vs. control; data are represented as median with interquartile range (25^th^; 75^th^); each group *n *= 6

IL-6, interleukin 6; p38-MAPK, p38-mitogen-activated protein kinases; rhAT, recombinant human antithrombin III; TNF-α, tumor necrosis factor alpha.

Western blots performed in myocardial tissues showed higher concentrations of p38-MAPK in the control vs. the sham group (*P *= 0.028, Table 3). In addition, there was an increase in myocardial concentrations of the inflammatory cytokines TNF-α and IL-6 in control vs. sham animals (*P *<0.05 each, Table 3). Contrarily, in the rhAT group concentrations of p38-MAPK (*P *= 0.01) as well as TNF-α (*P *= 0.046) and IL-6 (*P *= 0.038), were lower than in control animals.

## Discussion

The major findings of the present study are that 1) burn and smoke inhalation injury was associated with myocardial dysfunction and inflammation, and that 2) a continuous infusion of rhAT attenuated the impairment of myocardial contractility, 3) reduced myocardial inflammation, 4) limited NO production, and 5) reversed systemic fluid accumulation 6) without compromising plasmatic coagulation in a clinically relevant ovine model.

Burn- and smoke-induced myocardial dysfunction is typically characterized by an impaired left ventricular contractility [1,20]. In the present study, a reduced myocardial function in control animals was evidenced by lower SVI and LVSWI as compared to BL and sham animals. Although no direct measurement of contractility was performed, the Starling-based relation of LVSWI with the individual preload at this time (LAP) highly suggests an impaired myocardial contractility in the control vs. sham group. Notably, the time courses for SVI and HR in control animals nicely demonstrate the "two-hit" character of the injury [21]: the first one, immediately following the injury, is caused by the burn-induced shock and the associated systemic inflammatory response syndrome (SIRS). The second one is most probably attributed to an inflammatory response to the smoke inhalation usually occurring 24 h post injury [21,22].

As a compensatory mechanism, HR increased in control animals thereby maintaining cardiac output. However, myocardial oxygen consumption, as suggested by the (heart) rate-(mean arterial) pressure product [19], was consistently higher than in sham animals. In the acute phase of the injury, this increased myocardial oxygen consumption and the impairment of myocardial function potentially aggravate burn-induced shock as well as the associated SIRS, thereby eventually promoting multiple organ failure. Previously, burn- and smoke-induced myocardial dysfunction was thought to be transient and to resolve after successful treatment of the originating trauma [2]. Meanwhile, there is convincing evidence that it also influences long-term outcome. An increased HR and myocardial oxygen consumption, for example, were found to be present even three years after burn trauma in children [5,23]. Hypovolemia as a cause of reduced SVI and increased HR in the present study seems unlikely within the first 24 h, because in this case ventricular filling pressures as well as mean arterial pressure and cardiac index would have been decreased in control animals.

rhAT infusion markedly improved contractility, as suggested by higher SVI and LVSWI values as compared to control animals. In accordance with these findings, HR and myocardial oxygen consumption were significantly lower in rhAT-treated than in control animals. Whereas beneficial effects of AT on pulmonary gas exchange [10] and wound healing [24] in patients suffering from burn and smoke inhalation have been reported before, this is the first time that a therapeutic effect of AT on myocardial function is described.

Since "cardiac molecular signalling after burn trauma" involves several inflammatory pathways [7], the anti-inflammatory effects of rhAT represent a potential mechanism of action. As part of the burn- and smoke-induced SIRS, there is a pronounced activation of neutrophils leading to transendothelial migration into the organs and promotion of inflammatory cascades, like NO production and p38-MAPK pathway activation [6-8]. Neutrophil accumulation in heart tissues was suggested by an increase in myeloperoxidase [25] in control vs. sham animals that was attenuated by rhAT. This finding is well explained by the interaction of AT with the syndecan-4 receptor resulting in reduced neutrophil activation and chemotaxis [9,26].

In addition, activation of the p38-MAPK cascade plays a central role in burn-induced myocardial dysfunction [6,7,27-29]. It regulates apoptosis of cardiomyocytes in the early stage after severe burns [6,28] and promotes inflammation by increasing the production of TNF-α and downstream cytokines like IL-6 [1,6,27], both being significantly increased in the control vs. the sham group in the present study. Inhibition of p38-MAPK has been shown to improve myocardial function after burns *in vitro *[29] and in rats [27]. An inhibitory effect of AT on the p38-MAPK pathway, as suggested in the present study, is supported by an experiment on acute lung injury in rats [11]. Interestingly, the beneficial effects of AT in the latter study were abolished, if concomitant heparin was infused. Since heparin is known to block the interaction of AT with the syndecan-4 receptor [30], this mechanism may be involved in the reduction of p38-MAPK via AT. This hypothesis, however, requires further verification in future studies.

In accordance with the changes in p38-MAPK expression, concentrations of TNF-α and IL-6 were lower in rhAT-treated than in control animals. TNF-α and IL-6 have both been repeatedly reported to correlate with cardiac contraction and relaxation deficits after burn trauma [4,31,32] but also in heart failure patients [33]. Myocardial secretion of TNF-α may be directly responsible for an impaired myocardial contractility, reduced ejection fraction and biventricular dilation [7]. One mechanism of TNF-α-mediated cardiodepression includes the induction of NO synthesis [34]. The critical role of NO in burn- and smoke-induced myocardial dysfunction [8,35] is supported by the biphasic increase in NOx plasma levels that almost reflect the changes in SVI in control animals.

Vascular leakage, another hallmark of burn and smoke inhalation injury, was evidenced by marked decreases in protein concentration and oncotic pressures in injured animals. In this context, dilution due to over-resuscitation can be excluded, since hematocrit was maintained or even increased. Control animals progressively accumulated about 4,000 mL of fluid during the study period. rhAT-treated animals also had an initial drop in protein concentration and oncotic pressure that was probably caused by an irreversible loss via the burn wound. However, rhAT prevented a progression of protein leakage and reversed the initial fluid accumulation resulting in a fluid balance similar to sham animals at 48 h. The clinical relevance of this finding is emphasized by the correlation of fluid accumulation with negative outcome not only in burned patients [36] but also in critical ill patients in general [37]. Since all animals received the same amount of fluid (after adjustment for the individual body weight) and there were no differences in renal function (creatinine clearance) between groups, these findings suggest a reduction in vascular leakage by rhAT. This hypothesis is supported by several experimental studies reporting beneficial effects of AT on endothelial function. Potential mechanisms of action include interactions with the syndecan-4 receptor [9,26], preservation of the endothelial glycocalyx [38] and inhibition of endothelial cell activation [39]. The remaining difference in total urine output between sham and rhAT-treated animals may be explained by the additional fluid loss due to evaporation over the burn wound in the injured rhAT group.

The clinical applicability of the presented therapeutic approach is potentially limited by the early start of the infusion, the availability and unwanted side effects of AT. However, starting AT infusion one hour post-injury may be feasible, because contrary to most other syndromes patients with burn and smoke inhalation injury are transported to the hospital immediately and can be diagnosed rapidly. In addition, the recombinant preparation of AT ensures independence from blood donations and a reduced risk of transmitted infections. Finally, based on the present results, there seems to be no impairment of plasmatic coagulation with the low dose of 6 IU/kg/h rhAT.

This study has some limitations that we want to acknowledge. Due to the "two hit" character of the experimental model it is hard to differentiate the effects of rhAT on the burn-induced changes and the symptoms caused by smoke inhalation. However, the combined injury is of high clinical relevance. Another limitation is the estimation of LVSWI by standard formulas and the use of a correlation of LVSWI and LAP as an index of myocardial contractility, because inotropy should be assessed by load independent measures. However, these are only available by echocardiography. To avoid the compromising effects of anesthetics on myocardial function, the animals were awake during the experiment necessitating a ventilation regime that differed from the recommendation of the acute respiratory distress syndrome (ARDS) network. Finally, the smoke arising from house or industrial fires contains a multitude of toxic gases potentially resulting in differences in lung injury compared with the cotton smoke tested in the present study.

## Conclusions

In summary, this is the first study demonstrating therapeutic effects of rhAT substitution on myocardial dysfunction and inflammation as well as systemic fluid accumulation following burn and smoke inhalation injury in a clinically relevant animal model. Based on these findings, the supplementation of rhAT may represent a potential therapeutic approach for burn- and smoke inhalation-induced cardiovascular dysfunction.

## Key messages

• Combined burn and smoke inhalation injury is associated with a marked myocardial dysfunction. To compensate for the reduced stroke volume, heart rate and eventually myocardial oxygen consumption are increased.

• Impairment of myocardial function after burn and smoke inhalation injury is characterized by an activation of the p38-mitogen-activated protein kinase pathway, myocardial neutrophil accumulation and increased nitric oxide production.

• The therapeutic infusion of recombinant human antithrombin restored myocardial function to the level of sham (not injured) animals, resulting in higher stroke volumes and reduced myocardial oxygen consumption.

• The therapeutic infusion of recombinant human antithrombin reversed cumulative systemic fluid accumulation back to the level of sham animals at 48 h.

• The attenuated inflammatory response, as suggested by a reduced activation of the p38-mitogen-activated protein kinase pathway resulting in lower cytokine release, less myocardial neutrophil accumulation and less nitric oxide production, presents a potential mechanism of action.

## Abbreviations

ARDS: acute respiratory distress syndrome; AT: antithrombin III; BL: baseline; CVP: central venous pressure; HR: heart rate; IL-6: interleukin-6; LAP: left atrial pressure; LVSWI: left ventricular stroke work index; NO: nitric oxide; NOx: nitrates and nitrites; p38-MAPK: p38-mitogen-activated protein kinase; rhAT: recombinant human antithrombin III; SIRS: systemic inflammatory response syndrome; SVI: stroke volume index; TNF-α: tumor necrosis factor-alpha

## Competing interests

The authors declare that they have no competing interests.

## Authors' contributions

SR designed and performed the experiment, summarized and analyzed the data, and wrote the manuscript. YY, EB, LES, CJ and YZ performed the experiment, and summarized the data and edited the manuscript. LDT and DLT designed the experiment, analyzed the data and edited the manuscript. RAC performed the experiment, analyzed the data and edited the manuscript. PE designed and performed the experiment, analyzed the data and edited the manuscript. All authors read and approved the final manuscript.

## Supplementary Material

Additional file 1 **Supplemental file (word): detailed Materials and methods**. This file provides detailed information about the instrumentation, mechanical ventilation, hemodynamic monitoring, Western blots as well as laboratory and immunohistochemical analyses. Click here for file

Additional file 2 **Supplemental file (word): additional data**. This file provides additional data that may be of interest for the reader, but where not relevant in respect to the message of the study. Click here for file
