# Supplementary material for: Antithrombin attenuates myocardial dysfunction and reverses systemic fluid accumulation following burn and smoke inhalation injury: a randomized, controlled, experimental study
Source: Crit Care. 2013 May 11;17(3):R86. doi: 10.1186/cc12712 (PMC3706920; doi:10.1186/cc12712)
Supplement: Additional file 1 — Supplemental file (word): detailed Materials and methods. This file provides detailed information about the instrumentation, mechanical ventilation, hemodynamic monitoring, Western blots as well as laboratory and immunohistochemical analyses. [file cc12712-S1.DOC]

**Antithrombin attenuates myocardial dysfunction and reverses systemic fluid accumulation following burn and smoke inhalation injury:**

**a randomized, controlled, experimental study**

Sebastian Rehberg; Yusuke Yamamoto; Eva Bartha; Linda E Sousse; Collette Jonkam; Yong Zhu; Lillian D Traber; Robert A Cox; Daniel L Traber; Perenlei Enkhbaatar

**Detailed methods**

**Methods**

*Instrumentation and Surgical Procedures*

The ovine model of combined burn and smoke inhalation injury has been described in detail before [1, 2]. In brief, after induction of anaesthesia with i.m. injection of ketamine (KetaVedTM, Phoenix Scientific Inc., St. Joseph, MO, USA), eighteen adult female sheep were weighed and instrumented for chronic haemodynamic monitoring under deep anaesthesia (isoflurane, Abbott Lab., Abbott Park, IL, USA) with a right femoral artery catheter (18-GA, 36 inches; Parke-Davis, Sandy, UT, USA) and a Swan-Ganz thermal dilution catheter (REF 131F7; Edwards Lifesciences LLC, Irvine, CA, USA). In addition, the left atrium was cannulated (Silastic catheter, Dow Corning, Midland, MI, USA) through a thoracotomy incision in the sixth intercostal space. All intravascular catheters were connected to pressure transducers with continuous flushing devices (model PX3X3, Baxter-Edwards Critical Care, Irvine, CA, USA). The animals were then allowed to recover for one week with free access to water and food. Buprenorphine (0.3 mg) on a regular basis and whenever needed was used for analgesic therapy. One day before the experiment had begun, the animals were fasted, but had free access to water.

*Mechanical ventilation*

All animals were mechanically ventilated (Servo Ventilator 900C, Siemens-Elema, Sweden) with a tidal volume of 15 mL·kg^-1^ and a positive end-expiratory pressure of 5 cmH_2_O throughout the entire experiment. These ventilator settings are in accordance with previous studies using the same animal model [3, 4]. In this context, it is important to consider that the pulmonary compliance and dead space in sheep are higher than in humans. Therefore, higher tidal volumes than in humans are required to guarantee adequate ventilation [5]. In addition, the animals were not anaesthetized or paralyzed following the injury. As a consequence, low-tidal volume ventilation, as suggested by the ARDS network, was not possible in the awake animals. The inspiratory oxygen fraction was set at 100% for the first 3h post injury, and was then adjusted to maintain oxygenation (arterial oxygen saturation >90%, partial pressure of oxygen >90 mmHg), whenever possible. The respiratory rate was adjusted according to individual blood gas analyses to ensure normocapnia.

*Haemodynamic Monitoring*

Haemodynamic measurements were performed at BL, 3h, 6h, 9h, 12h, 15h, 18h, 21h, and 24h. Mean arterial pressure, central venous pressure, left atrial pressure and heart rate were continuously recorded on a haemodynamic monitor (model 7830A; Hewlett Packard, Santa Clara, CA). Cardiac output was measured in triplicate with the thermodilution technique using 10 mL of ice-cold normal saline as indicator. Cardiac index, stroke volume index, systemic vascular resistance index, left and right ventricular stroke work index as well as indexes of oxygen delivery, consumption and extraction were calculated using standard equations [6]. The product of heart rate and mean arterial pressure was calculated as an index of myocardial oxygen consumption [7]. Since sheep do not perspire and the inhaled gases were humidified, fluid balance was calculated as the difference of fluid input and urinary output [8]. During the experiment, the animals had free access to food, but not to water to accurately determine fluid intake.

*Laboratory analyses*

Arterial and mixed venous blood gas samples were analyzed for gas tensions and oxygen saturation using a blood gas analyzer (GEM Premier 3000, Instrumental Laboratory, Lexington, MA, USA) at BL, 3h, 6h, 12h, 18h, 24h, 36h, and 48h. For the following analyses, blood was sampled under sterile conditions at BL, 6h, 12h, 18h, 24h, 36h, and 48h. Haematocrit was measured by blood centrifugation in heparinized microhaematocrit capillary tubes (Fisher Brand, Pa, USA). Plasma protein concentration was measured with a refractometer (National Instrument, Baltimore, MD, USA). Oncotic pressure were determined through a semipermeable membrane in a colloid osmometer (model 4420; Wescor, Logan, UT, USA). Citrated blood samples were centrifuged, and plasma samples frozen at -80°C for determination of antithrombin plasma levels (antithrombin assay kit, S-2765, Diagnostica Stago, Parsippany, NJ, USA) and nitric oxide concentration by measuring the intermediate and end products according to the manufacturer’s protocol (nitrates/nitrites, Colorimetric Assay Kit, Cayman Chemicals Company, Ann Arbor, MI, USA) at a later time point. At BL, 12h, 24h, 36h, and 48h variables of plasmatic coagulation, namely activated clotting time, prothrombin time and activated partial thromboplastin time, were determined by Hemochron model 801 (International Technidyne Corp., Edson, NJ, USA). In addition, creatinine concentration was measured in the plasma and the urine at BL, 12h, 24h, 36h, and 48h.

*Immunohistochemical Analysis and Western blots*

Myocardial samples from the left ventricle (anterior wall) were snap frozen in liquid nitrogen, stored at -80°C and used for quantification of myeloperoxidase activity, phospho p38- Mitogen-Activated Protein Kinase (p38-MAPK), tumor necrosis factor alpha (TNF-alpha) and interleukin-6 (IL-6). Myeloperoxidase activity was determined using a commercially available assay (Myeloperoxidase Activity Assay, Northwest Life Science Specialties, Vancouver, Canada) according to the manufacturer’s protocol.

For western blot analyses, fifty milligrams of heart samples were homogenized in ice-cold 50 mM Tris-buffer, pH 8.0 (containing protease and phosphatase inhibitor cocktail 1:100 (Roche Applied Science, Indianapolis, IN, USA) and 50 mM sodium vanadate (Sigma-Aldrich Co., St. Louis, MO, USA)). After homogenization, samples were harvested in sodium dodecyl sulfate polyacrylamide gel electrophoresis sample buffer (NuPage; Invitrogen, Carlsbad, CA, USA). Proteins were separated on 4% to 12% sodium dodecyl sulfate polyacrylamide gels. After blocking (5% nonfat dry milk in phosphate-buffered saline), membranes were probed overnight at 4 °C with recognizing the following antigens: IL-6 (1:1000; Abcam, Cambridge, MA, USA), p38 MAPK Thr180-Gly-Tyr182 (1:1000; Cell Signaling Technology, Danvers, MA, USA), TNF-alpha (1:1000; Abcam, Cambridge, MA, USA), and actin (1:10000; Santa Cruz Biotechnology Inc, Santa Cruz, CA, USA). On the next day, membranes were washed three times for 15 min in phosphate buffered saline containing 0.5% Tween before addition of anti-rabbit or anti-mouse (according to the manufacturers` instructions) horseradish peroxidase-conjugated secondary antibody (1:3000; Southern Biotech, Birmingham, AL, USA). The antibody-antigen complexes were visualized by enhanced chemiluminescence method by Syngene gel documentation system (Syngene, Frederick, MD, USA). The results were quantified by GeneTools from Syngene.

**References**

1. Cox RA, Jacob S, Oliveras G, Murakami K, Enkhbaatar P, Traber L, Schmalstieg FC, Herndon DN, Traber DL, Hawkins HK: **Pulmonary expression of nitric oxide synthase isoforms in sheep with smoke inhalation and burn injury**. *Exp Lung Res* 2009, **35**:104-118.

2. Enkhbaatar P, Esechie A, Wang J, Cox RA, Nakano Y, Hamahata A, Lange M, Traber LD, Prough DS, Herndon DN, Traber DL: **Combined anticoagulants ameliorate acute lung injury in sheep after burn and smoke inhalation**. *Clin Sci (Lond)* 2008, **114**:321-329.

3. Lange M, Connelly R, Traber DL, Hamahata A, Cox RA, Nakano Y, Bansal K, Esechie A, von Borzyskowski S, Jonkam C, Traber LD, Hawkins HK, Herndon DN, Enkhbaatar P: **Combined neuronal and inducible nitric oxide synthase inhibition in ovine acute lung injury**. *Crit Care Med* 2009, **37**:223-229.

4. Enkhbaatar P, Connelly R, Wang J, Nakano Y, Lange M, Hamahata A, Horvath E, Szabo C, Jaroch S, Holscher P, Hillmann M, Traber LD, Schmalstieg FC, Herndon DN, Traber DL: **Inhibition of neuronal nitric oxide synthase in ovine model of acute lung injury**. *Crit Care Med* 2009, **37**:208-214.

5. Lange M, Traber DL, Enkhbaatar P: **The authors reply**. *Crit Care Med* 2011, **40**:356-357.

6. Westphal M, Sielenkamper AW, Van Aken H, Stubbe HD, Daudel F, Schepers R, Schulte S, Bone HG: **Dopexamine reverses the vasopressin-associated impairment in tissue oxygen supply but decreases systemic blood pressure in ovine endotoxemia**. *Anesth Analg* 2004, **99**:878-885, table of contents.

7. Gobel FL, Norstrom LA, Nelson RR, Jorgensen CR, Wang Y: **The rate-pressure product as an index of myocardial oxygen consumption during exercise in patients with angina pectoris**. *Circulation* 1978, **57**:549-556.

8. Westphal M, Enkhbaatar P, Schmalstieg FC, Kulp GA, Traber LD, Morita N, Cox RA, Hawkins HK, Westphal-Varghese BB, Rudloff HE, Maybauer DM, Maybauer MO, Burke AS, Murakami K, Saunders F, Horvath EM, Szabo C, Traber DL: **Neuronal nitric oxide synthase inhibition attenuates cardiopulmonary dysfunctions after combined burn and smoke inhalation injury in sheep**. *Crit Care Med* 2008, **36**:1196-1204.
