# Supplementary material for: Antithrombin attenuates myocardial dysfunction and reverses systemic fluid accumulation following burn and smoke inhalation injury: a randomized, controlled, experimental study
Source: Crit Care. 2013 May 11;17(3):R86. doi: 10.1186/cc12712 (PMC3706920; doi:10.1186/cc12712)
Supplement: Additional file 2 — Supplemental file (word): additional data. This file provides additional data that may be of interest for the reader, but where not relevant in respect to the message of the study. [file cc12712-S2.DOC]

**Antithrombin attenuates myocardial dysfunction and reverses systemic fluid accumulation following burn and smoke inhalation injury:**

**a randomized, controlled, experimental study**

Sebastian Rehberg; Yusuke Yamamoto; Eva Bartha; Linda E Sousse; Collette Jonkam; Yong Zhu; Lillian D Traber; Robert A Cox; Daniel L Traber; Perenlei Enkhbaatar

**Additional data**

**Table S1** **Variables of pulmonary gas exchange, pH, ventilation, global oxygen transport and plasmatic coagulation**

| **Variable** | **Time point** | **sham** | **control** | **rhAT** |
| --- | --- | --- | --- | --- |
| SaO_2_ | BL | 92 [90;92] | 94 [93;95] | 93 [92;93] |
| (%) | 6h | 93 [93;94] | 95 [94;96] | 93 [93;94] |
|  | 12h | 92 [91;93] | 93 [92;94] | 93 [93;97] |
|  | 24h | 93 [92;93] | 86 [79;89]*^#^ | 91 [91;92] |
|  | 36h | 93 [92;93] | 89 [88;89]*^#^ | 89 [82;93] |
|  | 48h | 93 [92;93] | 87 [85;90]*^#^ | 93 [90;94] |
| PaO_2_/FiO_2_ | BL | 502 [476;552] | 507 [467;538] | 523 [502;533] |
| (mmHg) | 6h | 516 [472;559] | 510 [408;520] | 531 [474;541] |
|  | 12h | 510 [500;517] | 452 [340;462]*^#^ | 555 [399;588] |
|  | 24h | 548 [537;551] | 144 [123;178]*^#^ | 396 [202;457]*^#^**^†^** |
|  | 36h | 512 [495;529] | 79 [79;97]*^#^ | 201 [110;310]*^#^**^†^** |
|  | 48h | 526 [510;557] | 83 [73;93]*^#^ | 164 [119;266]*^#^**^†^** |
| PaCO_2_ | BL | 39 [38;40] | 39 [36;41] | 41 [35;45] |
| (mmHg) | 6h | 29 [27;34]* | 30 [24;33]* | 30 [27;30]* |
|  | 12h | 32 [29;36]* | 28 [27;29]* | 28 [28;29]* |
|  | 24h | 33 [32;34]* | 30 [28;33]* | 30 [27;32]* |
|  | 36h | 30 [29;31]* | 41 [36;67]^#^ | 32 [29;46] |
|  | 48h | 29 [27;33]* | 52 [40;70]^#^ | 35 [33;46] |
| pH | BL | 7.49 [7.47;7.50] | 7.51 [7.50;7.52] | 7.48 [7.47;7.52] |
|  | 6h | 7.53 [7.47;7.56] | 7.57 [7.54;7.66]* | 7.57 [7.57;7.59]* |
|  | 12h | 7.47 [7.44;7.49] | 7.53 [7.50;7.54] | 7.52 [7.51;7.53] |
|  | 24h | 7.47 [7.44;7.50] | 7.54 [7.44;7.56] | 7.55 [7.52;7.56] |
|  | 36h | 7.50 [7.46;7.50] | 7.44 [7.32;7.48]* | 7.50 [7.43;7.51] |
|  | 48h | 7.46 [7.45;7.49] | 7.33 [7.25;7.41]*^#^ | 7.48 [7.39;7.51] |
| Shunt fraction | BL | 19 [18;21] | 15 [12;19] | 17 [12;23] |
| (%) | 6h | 15 [14;16]* | 11 [9;13] | 11 [11;14] |
|  | 12h | 19 [14;19] | 18 [17;18] | 15 [5;16] |
|  | 24h | 14 [12;19] | 34 [28;45]*^#^ | 17 [17;31] |
|  | 36h | 14 [13;18] | 44 [36;52]*^#^ | 19 [15;32]**^†^** |
|  | 48h | 14 [13;15]* | 44 [42;49]*^#^ | 31 [25;36]*^#^**^†^** |
| Respiratory rate | BL | 20 [20;20] | 20 [20;20] | 20 [20;20] |
| (breaths∙min^-1^) | 6h | 20 [20;20] | 20 [20;22] | 20 [20;20] |
|  | 12h | 20 [20;20] | 20 [16;22] | 20 [20;20] |
|  | 24h | 20 [20;20] | 22 [20;25] | 20 [20;20] |
|  | 36h | 20 [20;20] | 28 [23;36]*^#^ | 21 [18;36] |
|  | 48h | 20 [20;20] | 40 [32;41]*^#^ | 24 [20;38] |
| Peak pressure | BL | 19 [17;20] | 18 [17;21] | 19 [19;22] |
| (cmH_2_O) | 6h | 18 [18;18] | 19 [18;20] | 19 [18;21] |
|  | 12h | 19 [18;19] | 20 [20;23] | 20 [20;22] |
|  | 24h | 21 [17;24] | 31 [27;37]*^#^ | 22 [21;23]**^†^** |
|  | 36h | 18 [17;20] | 39 [36;41]*^#^ | 24 [21;35]^#^**^†^** |
|  | 48h | 19 [18;20] | 40 [37;43]*^#^ | 27 [24;33]*^#^**^†^** |
| Plateau pressure | BL | 18 [16;18] | 16 [15;19] | 17 [16;18] |
| (cmH_2_O) | 6h | 16 [16;17] | 16 [15;16] | 16 [15;19] |
|  | 12h | 17 [16;18] | 18 [18;19] | 17 [16;21] |
|  | 24h | 18 [16;20] | 27 [24;30]*^#^ | 18 [16;21]**^†^** |
|  | 36h | 16 [14;19] | 39 [33;39]*^#^ | 22 [18;32]**^†^** |
|  | 48h | 16 [16;17] | 39 [34;43]*^#^ | 25 [21;32]*^#^**^†^** |
| DO_2_I | BL | 758 [549;772] | 657 [542;726] | 720 [635;823] |
| (mL∙min^-1^∙m^-2^) | 6h | 705 [655;861] | 733 [610;780] | 636 [615;853] |
|  | 12h | 780 [762;886] | 724 [573;860] | 766 [698;796] |
|  | 24h | 779 [705;853] | 584 [545;633]^#^ | 822 [683;901]**^†^** |
|  | 36h | 692 [647;706] | 646 [610;799] | 643 [598;842] |
|  | 48h | 660 [614;753] | 719 [654;809] | 642 [501;787] |
| VO_2_I | BL | 270 [231;298] | 224 [212;271] | 277 [204;331] |
| (mL∙min^-1^∙m^-2^) | 6h | 271 [243;357] | 328 [297;389]* | 323 [290;375] |
|  | 12h | 318 [296;341] | 284 [229;319] | 331 [292;347] |
|  | 24h | 314 [299;365] | 239 [226;310] | 362 [333;393]**^†^** |
|  | 36h | 275 [259;304] | 261 [261;283] | 354 [290;367]**^†^** |
|  | 48h | 262 [254;313] | 336 [262;387] | 290 [211;325] |
| O_2_ER | BL | 40 [39;40] | 38 [37;39] | 37 [33;48] |
| (%) | 6h | 40 [36;42] | 46 [45;49]*^#^ | 48 [43;52] |
|  | 12h | 39 [36;41] | 39 [38;42] | 41 [40;43] |
|  | 24h | 42 [37;48] | 42 [39;46] | 45 [37;50] |
|  | 36h | 41 [39;45] | 41 [33;47] | 43 [41;47] |
|  | 48h | 42 [39;47] | 37 [31;43] | 42 [35;46] |
| ACT | BL | 140 [135;158] | 140 [132;155] | 133 [132;141] |
| (sec) | 6h | / | / | / |
|  | 12h | 155 [141;164] | 159 [139;166] | 137 [134;169] |
|  | 24h | 152 [132;161] | 142 [124;159] | 159 [132;172] |
|  | 36h | 163 [125;166] | 149 [145;158] | 140 [123;147] |
|  | 48h | 140 [127;168] | 132 [125;148] | 133 [132;147] |
| PT | BL | 72 [68;76] | 80 [78;86] | 73 [60;81] |
| (sec) | 6h | / | / | / |
|  | 12h | 63 [62;67] | 95 [87;102]^#^ | 89 [82;106] ^#^ |
|  | 24h | 68 [64;69] | 88 [72;93]^#^ | 87 [74;94] ^#^ |
|  | 36h | 67 [62;72] | 110 [107;128]*^#^ | 100 [86;116]*^#^ |
|  | 48h | 67 [66;81] | 101 [89;109]*^#^ | 84 [79;90] |
| aPTT | BL | 124 [120;130] | 115 [111;119] | 114 [112;120] |
| (sec) | 6h | / | / | / |
|  | 12h | 121 [117;131] | 118 [104;124] | 119 [112;138] |
|  | 24h | 131 [91;139] | 124 [114;136] | 116 [107;131] |
|  | 36h | 131 [122;144] | 119 [114;124] | 118 [113;130] |
|  | 48h | 114 [102;119] | 125 [113;130] | 112 [111;127] |
| Platelets | BL | 505 [481;713] | 682 [431;991] | 624 [534;697] |
| (1000∙μL^-1^) | 6h | / | / | / |
|  | 12h | 532 [453;698] | 639 [424;726] | 582 [570;805] |
|  | 24h | 641 [352;869] | 730 [517;866] | 699 [558;758] |
|  | 36h | 444 [357;702] | 534 [490;853] | 760 [439;824] |
|  | 48h | 479 [317;831] | 567 [421;637] | 645 [373;761] |

*,***p*<0.05 vs. BL; ^#^**,***p*<0.05 vs. sham; ^†^,*p*<0.05 vs. control; data are represented as median with interquartile range [25^th^; 75^th^];** **each group n=6**

**ACT, activated clotting time; aPTT, activated partial thromboplastin time; BL, baseline; DO_2_I, oxygen delivery index; FiO_2_, inspiratory oxygen fraction; O_2_ER, oxygen extraction rate; PaCO_2_, partial pressure of carbon dioxide; PaO_2_, partial pressure of oxygen; pH, potentia hydrogenii; PT, prothrombin time; rhATIII, recombinant human antithrombin III; SaO_2_, arterial oxygen saturation; VO_2_I, oxygen consumption index**
